# First Detection of SARS-CoV-2 B.1.1.7 Variant of Concern in an Asymptomatic Dog in Spain

**DOI:** 10.3390/v13071379

**Published:** 2021-07-15

**Authors:** Sandra Barroso-Arévalo, Belén Rivera, Lucas Domínguez, José M. Sánchez-Vizcaíno

**Affiliations:** 1 VISAVET Health Surveillance Center, Animal Health Department, Complutense University of Madrid, 28040 Madrid, Spain; belenriv@ucm.es (B.R.); lucasdo@visavet.ucm.es (L.D.); jmvizcaino@ucm.es (J.M.S.-V.); 2 Department of Animal Health, Faculty of Veterinary, Complutense University of Madrid, 28040 Madrid, Spain

**Keywords:** SARS-CoV-2, variant of concern, dog, B.1.1.7, pet, reverse zoonosis

## Abstract

Natural SARS-CoV-2 infection in pets has been widely documented during the last year. Although the majority of reports suggested that dogs’ susceptibility to the infection is low, little is known about viral pathogenicity and transmissibility in the case of variants of concern, such as B.1.1.7 in this species. Here, as part of a large-scale study on SARS-CoV-2 prevalence in pets in Spain, we have detected the B.1.1.7 variant of concern (VOC) in a dog whose owners were infected with SARS-CoV-2. The animal did not present any symptoms, but viral loads were high in the nasal and rectal swabs. In addition, viral isolation was possible from both swabs, demonstrating that the dog was shedding infectious virus. Seroconversion occurred 23 days after the first sampling. This study documents the first detection of B.1.1.7 VOC in a dog in Spain and emphasizes the importance of performing active surveillance and genomic investigation on infected animals.

## 1. Introduction

Since December 2019, the severe acute respiratory syndrome coronavirus-2 (SARS-CoV-2) has spread worldwide, triggering one of the most challenging pandemics so far [1,2]. In the last months, several variants of this virus have been reported [3,4], some of which have raised additional concern due to increased transmissibility [5], higher rates of mortality [6], escape from immune system response [7], altered vaccine effectiveness [7,8] or failure in diagnosis tests [9]. These variants of concern (VOC) carry several genetic mutations, most of which are at the level of the spike (S) protein receptor-binding domain (RBD), which plays a key role in triggering virus entry into the cell, by binding to the cell host angiotensin-converting enzyme 2 (ACE-2) receptor. To date, four VOC have been described, including B.1.1.7 (also referred to as 20I/N501Y.V1), first detected in the United Kingdom (UK) [3]; B.1.351 (20J/N501Y.V2), first identified in South Africa [4]; P.1 (20I/N501Y.V3), detected in Brazil; and B.1.617 (G/452R.V3), recently identified in India. The origin of these variants remains unclear. Taking into account the susceptibility of several animal species to the virus [10] and the mechanisms underlying mutations [11], a zoonotic source cannot be dismissed. Indeed, a recent study based on an extensive phylogenetic assay has reported that *Mustelidae, Felidae* and especially *Canidae* families could be a possible host of the direct progenitor of variant B.1.1.7 [12]. Additionally, human-to-animal transmission of SARS-CoV-2 has been widely demonstrated under natural conditions [13,14,15,16], which may increase the mutation rate of the virus, due to viral adaptation to the host [17]. However, little is known about the potential pathogenicity and transmission rates of these variants in animal hosts. Therefore, surveillance of SARS-CoV-2 in animals and genetic investigation of viruses that have been isolated from infected pets is critical for understanding the transmission and evolution of the virus. Here, we document the detection of the B.1.1.7 VOC in a dog living with its infected owners, as part of a large-scale study of SARS-CoV-2 prevalence in pets in Spain. This is, to our knowledge, the first detection of this variant in a dog in Spain, which is a country with a high rate of SARS-CoV-2 infection. This outcome evidences that B.1.1.7 variant transmission from humans to animals can occur, and emphasizes the importance of studying the VOC spread from humans to animals.

## 2. Materials and Methods

### 2.1. Detection of SARS-CoV-2 Infection by Reverse Transcription-Quantitative PCR (RT-qPCR) and Virus Isolation

Nasal and rectal swabs were collected in DeltaSwab^®^ Virus 3 mL contained in viral transport media (VTM) (Deltalab S.L., Cataluña, Spain) using protocols approved by the Complutense University of Madrid’s ethics committee for animal experiments (project license 14/2020). RNA from these swabs was extracted using the KingFisher Flex System automated extraction instrument (Thermo Fisher, Waltham, MA, USA), with the MagMAX viral/pathogen nucleic acid isolation kit (Thermo Fisher), according to the manufacturer’s instructions. The detection of SARS-CoV-2 RNA was performed using the envelope protein (E)-encoding gene (Sarbeco) and two targets (IP2 and IP4) of the RNA-dependent RNA polymerase gene (RdRp) in an RT-qPCR protocol established by the World Health Organization, according to the guidelines that can be found at https://www.who.int/emergencies/diseases/novel-coronavirus-2019/technical-guidance/laboratory-guidance (accessed on 1 July 2020) [18].

Viral isolation was performed using the previously described methods in [16]. Briefly, specimens that tested positive for qRT-PCR were subjected to virus isolation in Vero E6 cells (ATCC^®^, Manassas, VA, USA). These cells were cultured in Gibco Roswell Park Memorial Institute (RPMI) 1640 medium with L-glutamine (Lonza Group Ltd., Basel, Switzerland) and supplemented with 100 IU/mL penicillin, 100 μg/mL streptomycin, and 10% fetal bovine serum (FBS) (Merck KGaA, Darmstadt, Germany) (growth medium). The cells were then seeded in 96-well culture plates and cultured at 37 °C with 5% CO_2_ for 24 to 48 h, after which they were inoculated with 10 μL of the direct sample contained in VTM (oronasal or rectal swabs). Mock-inoculated cells were used as negative controls. The cultured cells were maintained at 37 °C with 5% CO_2_, with a daily observation of CPE and cellular death. After 6 days, the cell cultures were frozen, thawed, and subjected to three passages with inoculations of fresh Vero E6 cell cultures with the lysates, as described above. SARS-CoV-2 molecular detection was performed by employing RT-qPCR on the supernatants from every passage in order to confirm the presence/absence of the virus in the cell culture and virus recovery by means of the decrease in Ct.

### 2.2. Neutralizing Antibody Detection by Virus Neutralization Test (VNT)

Sera were tested for neutralizing antibodies against SARS-CoV-2 employing a virus neutralization test (VNT). Briefly, the VNT was performed in duplicate in 96-well plates by incubating 25 μL of two-fold serially diluted sera with 25 μL of 100 TCID50/mL of the SARS-CoV-2 MAD6 isolate (kindly provided by Dr. Luis Enjuanes). The virus–serum mixture was incubated at 37 °C with 5% CO_2_. At 1 h post-incubation, 200 μL of Vero E6 cell (provided by the Carlos III Health Institute, Madrid, Spain) suspension was added to the virus–serum mixtures, and plates were incubated at 37 °C with 5% CO_2_. Neutralization titers were determined 3 days post-infection. The titer of a sample was recorded as the reciprocal of the highest serum dilution that provided at least 100% neutralization of the reference virus, as determined by the visualization of cytopathic effect (CPE).

### 2.3. Phylogenetic Analysis of SARS-CoV-2 Spike Protein by PCR Amplification and DNA Sequencing

The S protein genome from the nasal sample was sequenced using the primer walking approach with 6 primer pairs (Table 1), which were designed based on the reference genome of SARS-CoV-2 isolate Wuhan-Hu-1 (NC_045512.2). PCR was performed using the Platinum Green Hot Start PCR master mix (Invitrogen, Waltham, MA, USA) in reactions containing 15 μL of master mix, 1 μL of forward and reverse primers (20 μM), 0.5 μL of RT Kapa enzyme (Kapa Biosystem, Woburn, MA, USA), 0.4 μL of dNTP mix (Thermo Fisher Scientific, Waltham, MA, USA), 2 μL of RNA template, and 5.1 μL of RNAse- and DNase-free water. Reactions were subjected to RT activation (48 °C, 15 min), initial denaturalization (98 °C, 30 s) and 45 cycles of denaturation at 98 °C for 10 s, annealing at 57 °C for 30 s, extension at 72 °C for 30 s, and final extension at 72 °C for 7 min in a T3000 thermocycler (Biometra, Göttingen, Germany).

The amplified PCR products were analyzed using 1% agarose gel electrophoresis in 45 mM Tris borate (pH 8.0) and 2.5 mM EDTA (0.53 TBE) containing 0.5 mg/mL ethidium bromide; DNA products were visualized by transillumination with a long-wave UV lightbox. PCR products were purified using a PCR purification kit (Qiagen, Germantown, PA, USA), and >600 bp (excluding primers) were sequenced using the Sanger method on an ABI Prism 3730 (Applied Biosystems, Foster City, CA, USA). The sequencing primers were the same as those used for amplification. S sequence was edited and assembled into the S genome using MEGA 6 software [19]. The sequence was aligned with published S sequences from global initiative on sharing all influenza data (GISAID) using MUSCLE. Mutations were determined using the CoVsurver mutations app available on the GISAID website (https://www.gisaid.org/) (accessed on 1 July 2021). We gratefully acknowledge the various laboratories and contributors of GISAID for providing these SARS-CoV-2 sequences.

## 3. Results

The dog, a 14-year-old giant poodle male, tested positive in both nasal and rectal swabs for SARS-CoV-2 RNA detection by RT-qPCR on 24 March 2021, 7 days after its owners received a positive test result for COVID-19. The viral loads, based on PCR Ct values, were high (Table 2). The animal was asymptomatic according to the veterinary inspection, which included temperature measuring and overall clinical evaluation. Live virus isolation was possible from both the nasal and rectal swabs, with CPE noted in all passages, in which virus recovery was confirmed by using PCR. Two days after the first sampling (26 March 2021), the animal was resampled, once again showing positive results for both the nasal and rectal swabs. The swab samples showed negative results 23 days after the initial sampling (13 April 2021). Sera samples were also taken in every sampling and evaluated by VNT. While sera from first and second samplings tested negative for VNT, the serum from third sampling was positive, with a titer of 1/256.

SARS-CoV-2 S genome sequencing was successful from the first nasal swab (GenBank reference number: MZ299152). The sequence was identified as B.1.1.7 VOC by alignment in MEGA software using the MUSCLE algorithm. According to the CoVsurver mutations app, the sequence presented the nine nucleotide changes that are characteristic of this variant, as follows: two deletions (H69del, V70del) and seven mutations (N501Y, A570D, D614G, P681H, T716I, S982A, D1118H). In addition to the distinctive B.1.1.7 mutations, the S sequence also showed one deletion (Y144del) and two mutations (D138H and E619K).

## 4. Discussion

This is, to our knowledge, the first report of infection in a dog by the B.1.1.7 variant of SARS-CoV-2 in Spain, a country that currently presents a high prevalence of this variant [20]. The transmission of this variant from humans to their pets has also been described in the UK and the United States (US) [21,22], this being the third report documenting this event. Despite the low rate of SARS-CoV-2 infection in pets under natural conditions, VOCs can easily spread to animals who are exposed to contaminated environments. Herein, sequencing analysis was not performed from the owners’ samples, but they appear to be the most likely source of infection for the dog, since the animal was quarantined with them during their infection period. Dogs’ susceptibility has been demonstrated to be very low under both experimental and natural conditions [13,23,24]. This fact is supported by the low viral loads that have been detected in infected animals, together with the non-appearance of symptoms. However, the infection by the B.1.1.7 variant has been related to symptomatology, such as sneezing [22], or even cardiomyopathy [21]. In our study, a high viral load was detected, despite the dog not showing any clinical signs. Moreover, virus isolation was possible from both the nasal and rectal swabs. These facts may suggest that the animal was shedding infectious virus. Therefore, the dog may have been a source of infection for other pets, or even humans. Taking into account that restriction and quarantine measures in infected animals are not clearly established yet, animals infected by VOCs such as B.1.1.7 may be a risk for public health. As these variants are broadly distributed in human populations, and their transmission capacity seems to be higher than in the original virus, active surveillance should be conducted in animals, in order to prevent anthropozoonoses, and especially reverse zoonoses, which is a phenomenon that has already been demonstrated to occur with SARS-CoV-2 in the case of minks [25,26]. It is known that coronaviruses commonly tend to display a rapid evolution when they jump to different species [27]. In this sense, a recent study has analyzed the S genes and proteins of existing SARS-CoV-2 strains that were collected from animals, to find a possible direct progenitor of variant B.1.1.7. In this study, the authors suggested that the variant strains in humans could not have evolved into the early variant B.1.1.7, but they might have infected high-density, yet susceptible, animals (such as dogs), and adapted to these species through rapid mutation. Therefore, all these results are in line with public health recommendations and highlight the importance of isolating SARS-CoV-2-infected people from their pets [28], in order to prevent human-to-pet transmission. This is especially important in the case of infection with VOCs, whose transmission and pathogenicity characteristics in animals remain unknown.

## Figures and Tables

**Table 1:** Polymerase Chain Reaction (PCR) primers used for spike genome amplification based on the reference genome of SARS-CoV-2 isolate Wuhan-Hu-1 (NC_045512.2).

Primer Pairs	Forward Primer	Reverse Primer	Product Size
Spike-1	TGCGTCATCATCTGAAGCAT	CGAAAAACCCTGAGGGAGAT	973
Spike-2	GGACCTTGAAGGAAAACAGG	CCTGGAGCGATTTGTCTGA	708
Spike-3	CCGCATCATTTTCCACTTTT	CGCATATACCTGCACCAATG	903
Spike-4	GCACAGAAGTCCCTGTTGCT	GGTTGGCAATCAATTTTTGG	957
Spike-5	CTGCACTGTTAGCGGGTACA	GGCGGTCAATTTCTTTTTGA	933
Spike-6	ATGATCCTTTGCAACCTGAA	ATGATTTTGGAAGCGCTCTG	606

**Table 2:** Longitudinal SARS-CoV-2 test results for a pet dog from Madrid (Spain) that was confirmed for infection with the B.1.1.7 variant of concern (VOC).

Animal ID, Date of Sample Collection	RT-qPCR Ct Values for Swab Testing		Viral Neutralization Endpoint Titer	Viral Isolation
+D-7, 24.03.2021	Nasal swab	Rectal swab	Negative	Yes
	20.05	26.34		
+D-7, 26.03.2021	Nasal swab	Rectal swab	Negative	NP
	20.21	33.55		
+D-7, 13.04.2021	Nasal swab	Rectal swab	1/256	NA
	ND	ND		

ND: not detected; NP: not performed; NA: not applied.

## Data Availability

The data that support the findings of this study are available from the corresponding author upon reasonable request.
